# Controlled human wood smoke exposure: oxidative stress, inflammation and microvascular function

**DOI:** 10.1186/1743-8977-9-7

**Published:** 2012-03-27

**Authors:** Lykke Forchhammer, Peter Møller, Ingunn Skogstad Riddervold, Jakob Bønløkke, Andreas Massling, Torben Sigsgaard, Steffen Loft

**Affiliations:** 1 Section of Environmental Health, Department of Public Health, University of Copenhagen, Copenhagen, Denmark; 2 Department of Environmental and Occupational Medicine, School of Public Health, University of Aarhus, Aarhus, Denmark; 3 Department of Environmental Sciences, Aarhus University, Roskilde, Denmark; 4 Øster Farimagsgade 5, Build. 5, 2nd floor, Postbox 2099, DK-1014 Copenhagen, K, Denmark

**Keywords:** Wood smoke, Particulate matter, Inflammation, Oxidative stress, Endothelial dysfunction, DNA damage

## Abstract

**Background:**

Exposure to wood smoke is associated with respiratory symptoms, whereas knowledge on systemic effects is limited. We investigated effects on systemic inflammation, oxidative stress and microvascular function (MVF) after controlled wood smoke exposure.

**Methods:**

In a randomised, double-blinded, cross-over study 20 non-smoking atopic subjects were exposed at rest to 14, 220, or 354 μg/m^3 ^of particles from a well-burning modern wood stove for 3 h in a climate controlled chamber with 2 week intervals. We investigated the level of oxidatively damaged DNA, inflammatory markers and adhesion molecules before and 0, 6 and 20 h after exposure. Six h after exposure we measured MVF non-invasively by digital peripheral artery tonometry following arm ischemia.

**Results:**

The MVF score was unaltered after inhalation of clean air (1.58 ± 0.07; mean ± SEM), low (1.51 ± 0.07) or high (1.61 ± 0.09) concentrations of wood smoke particles in atopic subjects, whereas unexposed non-atopic subjects had higher score (1.91 ± 0.09). The level of oxidatively damaged DNA, mRNA of *ITGAL*, *CCL2*, *TNF*, *IL6*, *IL8*, *HMOX1*, and *OGG1 *and surface marker molecules ICAM1, ITGAL and L-selectin in peripheral blood mononuclear cells were not affected by inhalation of wood smoke particles.

**Conclusions:**

Exposure to wood smoke had no effect on markers of oxidative stress, DNA damage, cell adhesion, cytokines or MVF in atopic subjects.

## Background

Despite improvements in design and use of wood stoves, wood smoke is still an important local source of particulate matter (PM) in many communities [1]. Health effects and mechanisms of action related to exposure to wood smoke particles are less investigated than those associated with ambient PM from traffic related sources [2-4]. The mechanisms proposed to explain the adverse health effects of PM exposure include particle-induced oxidative stress, inflammation and genotoxicity [5-7]. Several *in vitro *studies of cultured cells have previously shown that wood smoke PM increased the expression and production of pro-inflammatory cytokines, oxidatively damaged DNA and oxidative stress [8-11]. A controlled exposure study of wood smoke particles in healthy humans showed minor effects related to oxidative stress and inflammation, including increased concentration of malondialdehyde and nitric oxide in exhaled breath condensate as well as altered coagulation factor levels in blood [12-14]. In the same study unaltered levels of oxidatively damaged DNA in peripheral blood mononuclear cells (PBMCs) was found, whereas there was increased mRNA expression of the DNA repair protein *oxoguanine glycosylase 1 *(*OGG1*) and larger urinary excretion of the repair product 8-oxo-7,8-dihydroguanine (8-oxoGua) suggesting that exposure to wood smoke particles was associated with enhanced DNA repair activity [15]. Recently, the results from a new study indicated virtually no effect on inflammation and oxidative stress in the airways after inhalation of relative large concentrations (224 ± 22 μg/m^3 ^for 3 h) of PM from a wood pellet burner [16]. It appears that wood smoke PM shows relatively limited effects measured by biomarkers in healthy subjects, but vulnerable subjects such as asthmatics or atopics, who are predisposed to allergy and constitute more than 20% of the Danish population [17], might be particularly sensitive to inhalation exposure of wood smoke PM [18]. Moreover, effects on vascular function associated with cardiovascular disease have been insufficiently addressed with respect to wood smoke PM, whereas exposure to ambient air and traffic generated PM is consistently associated with vascular disease and dysfunction [3,4,19]. The aim of this study was to investigate the effect on oxidative stress, systemic inflammation and microvascular function (MVF) after controlled exposure to wood smoke in atopic subjects. It has previously been shown that elderly persons had improved MVF after reduction of indoor particle exposure by filtration of recirculating air [20]. Similar effects of such an intervention have recently been shown among healthy individuals living in a wood smoke impacted community [21], whereas increased exposure to ambient air particles from a busy street had no effect on MVF in young and healthy subjects [22]. We assessed the level of oxidatively damaged DNA and the expression of *OGG1 *and *heme oxygenase 1 (HMOX1*) in PBMCs because these are sensitive endpoints for particle-induced oxidative stress [7]. The activation of these cells was assessed by expression of inflammatory genes including *chemokine (C-C-motif) ligand 2 *(*CCL2), interleukin 6 (IL6), interleukin 8 (IL8), tumor necrosis factor (TNF) *and surface markers including inter cellular adhesion molecule 1 (ICAM1), ITGAL integrin αL (antigen CD11A, lymphocyte function-associated antigen 1; α-polypeptide) and L-selectin.

## Results

**Figure 1:**
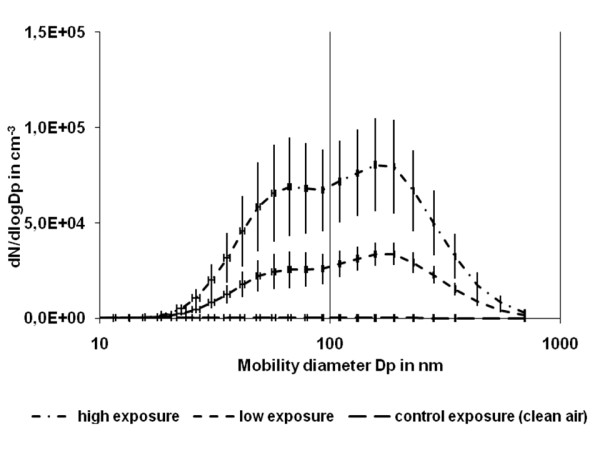
**Mean mobility particle number size distribution obtained during the sessions of different exposure types representing two size modes of particulate matter in the climate chamber**. The data are from [23] and the error bars are SEM.

The present study investigated the impact of different doses of wood smoke-derived PM_2.5 _(mean ± SD); a clean air exposure 14 ± 8 μg/m^3^, a relatively low concentration 220 ± 49 μg/m^3 ^and a relatively high concentration 354 ± 148 μg/m^3 ^(Table 1). The variation in observed particle number size distribution during the exposure sessions was considerable as illustrated by the error bars for 15 min values over the full exposure time of each exposure session type, depicted in Figure 1. Two size modes of particles with mean diameters of 67 nm and 157 nm were clearly visible at both exposure concentrations. There were high levels of polycyclic aromatic hydrocarbons (PAH) in PM collected at both exposure concentrations (Table 1).

**Table 1:** Levels of air pollutants measured during the exposure scenarios with clean air low and high level of wood smoke in the chambers

Measurement	Clean air	Low level exposure	High level exposure
CO level (ppm)	0 ± 0	9.85 ± 3.54	16.05 ± 4.74
PM_2.5 _stationary (μg/m^3^)	14 ± 8	220 ± 49	354 ± 148
Total particle (number/cm^3^)	222 ± 358	29112 ± 11883	71036 ± 46471
Chrysene + Triphenylene (μg/m^3^)	0.7 ± 0.6	284 ± 132	342 ± 284
Benzofluoranthenes (μg/m^3^)	0.3 ± 0.4	167 ± 127	196 ± 83
Benzo[e]pyrene (ng/m^3^)	0.2 ± 0.4	197 ± 134	248 ± 107
Benzo[a]pyrene (ng/m^3^)	0.2 ± 0.3	329 ± 249	325 ± 128
Perylene (ng/m^3^)	ND	43 ± 32	42 ± 18
Indeno(1,2,3-cd)pyrene (ng/m^3^)	0.1 ± 0.2	237 ± 173	249 ± 104
Dibenzo(a, h)anthrance (ng/m^3^)	ND	23 ± 12	25 ± 8
Benzo(ghi)perylene (ng/m^3^)	ND	180 ± 136	181 ± 81

Values are mean ± SD. (*ND*: Not detected)

**Figure 2:**
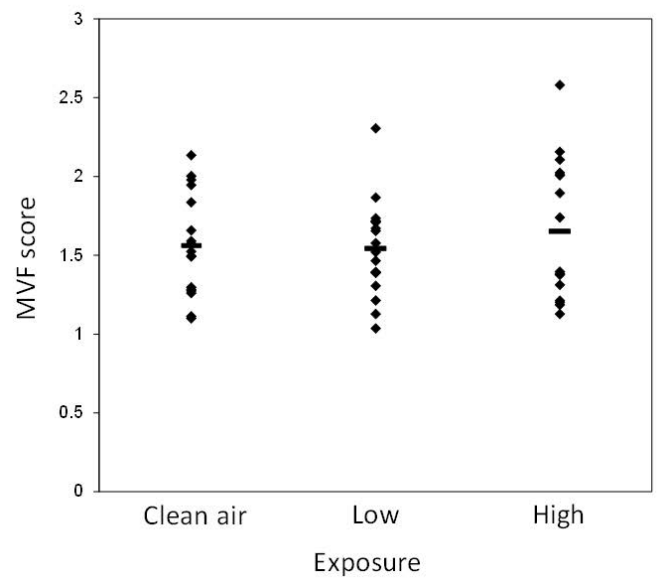
**Microvascular function (MVF) score 6 h after termination of each of the different exposures (n = 20)**. Each symbol corresponds to a person, whereas the bars represent the mean of each exposure. The mean ± SEM were 1.61 ± 0.09, 1.51 ± 0.09, 1.55 ± 0.08 in the subjects exposed to clean air, low and high content of wood smoke particles, respectively.

The results from the assessment of MVF are outlined in Figure 2. Two pulse wave tracings of MVF were not recorded due to instrument failure. We found no significant associations between the exposure and MVF score (*p = *0.78). The average relative change (95% confidence interval) in MVF from the value measured after clean air exposure was 2% (-9% to 13%), 5% (-5% to 16%) and 3% (-5% to 10%) after low and high level of wood smoke exposure and for the mean value after the two exposure levels, respectively. The overall average MVF score, mean ± SEM, was relatively low (1.56 ± 0.04) in this group of atopic subjects compared to our earlier observations in healthy subjects, elderly, and the threshold for abnormal MVF score. In our previous investigations we have assessed the MVF in the morning, whereas the same endpoint was obtained in the late afternoon in this wood smoke exposure study.

We carried out a small experiment on MVF in 8 healthy non-atopic subjects, who had their MVF measured on four occasions during one day. The mean ± SEM of the MVF score were 1.78 ± 0.17, 1.92 ± 0.10, 2.05 ± 0.28 and 1.88 ± 0.21 for the MVF measured in the morning (8.00-9.30 am), before lunch (10.00-11.30 am), after lunch (13.30-15.00 pm) and in the afternoon (15.30-17.30 pm). There was no effect on the time of the day on the MVF score (3% of the overall variation; *p *= 0.51), whereas there was substantial inter-individual variation (67% of the overall variation, *p *< 0.001). The remaining 30% of the total variation represents both the intra-individual and measurement variation.

**Figure 3:**
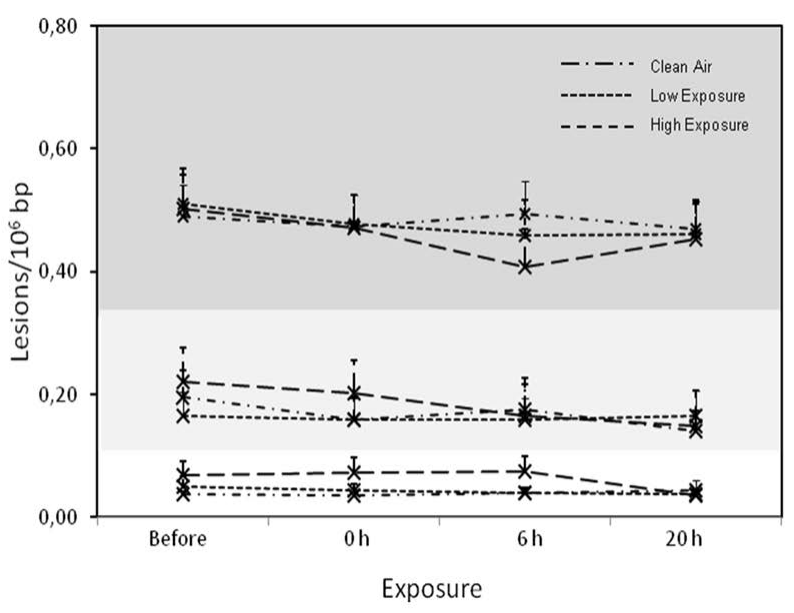
**Level of DNA damage in PBMCs measured 4 times during 24 h after each of the different wood smoke exposure scenarios (n = 20)**. The figure is divided in three sections. The dark grey section depicts the FPG sensitive sites. The light grey section depicts the EndoIII sensitive sites and the white section depicts the SB.

Figure 3 depicts the relationship between the levels of DNA damage detected by the comet assay in terms of strand break (SB), endonuclease III (EndoIII) and formamidopyrimidine DNA glycosylase (FPG) sensitive sites [24] and different exposure scenarios at different time points. The SBs measured by the alkaline comet assay represent unspecific DNA damage, whereas incubation of the DNA with EndoIII or FPG gives measurements of oxidatively damaged pyrimidines and purines bases, respectively. Wood smoke exposure had no significant effect on the level of SB (*p *> 0.09), EndoIII- (*p *> 0.12) or FPG sensitive sites (*p *> 0.89). A test of the statistical power showed that we would have been able to measure a difference of 0.14 lesions/10^6 ^bp between the groups with 17 subjects per group. This means that we would be able to measure a 23% change in the level of oxidatively damaged DNA. The level of DNA damage (mean ± SEM) in the reference control samples were 0.16 ± 0.03 lesions/10^6 ^bp (SB, n = 18) and 1.12 ± 0.10 lesions/10^6 ^bp (FPG sensitive sites, n = 18).

The mRNA levels according to exposure and time after exposure are given in Table 2. There were no significant effects of exposure on the mRNA levels of *IL6 *(*p *= 0.12), *IL-8 *(*p *= 0.37), *TNF *(*p *= 0.38), *CCL2 *(*p *= 0.96), *ITGAL *(*p *= 0.60), *OGG1 *(*p *= 0.58) or *HMOX1 *(*p *= 0.61). Table 2 also summarises that the expression of adhesion molecules of ICAM1 (*p *= 0.89), ITGAL (*p *= 0.51), and L-selectin (*p *= 0.07) on PBMCs by flow cytometry was not associated with the exposure to wood smoke.

**Table 2:** Mean ± SEM of gene expression (mRNA levels per 10^6 ^18 S mRNA) and the geometric mean ± SEM of adhesion molecule expression by FACS analysis in peripheral blood mononuclear cells (PBMCs) at four times after each of the three exposure scenarios

		Clean air			Low level wood smoke exposure				High level wood smoke exposure			
	
	Before	0 h	6 h	20 h	Before	0 h	6 h	20 h	Before	0 h	6 h	20 h
Gene expression												
*CCL2*	0.08 ± 0.04	0.05 ± 0.02	0.06 ± 0.02	0.10 ± 0.05	0.15 ± 0.09	0.18 ± 0.10	0.12 ± 0.05	0.02 ± 0.02	0.06 ± 0.02	0.14 ± 0.09	0.16 ± 0.08	0.22 ± 0.11
*ITGAL*	52.9 ± 22.5	57.0 ± 28.4	70.2 ± 33.6	173 ± 108	206 ± 172	203 ± 135	81.3 ± 36.4	15.8 ± 3.99	72.7 ± 37.6	46.8 ± 22.9	49.6 ± 17.0	86.01 ± 51.7
*TNF*	75.1 ± 73.0	4.10 ± 1.64	4.31 ± 1.48	8.14 ± 2.86	7.43 ± 3.46	8.91 ± 3.74	4.78 ± 1.49	2.35 ± 1.60	3.61 ± 1.63	2.42 ± 0.99	59.0 ± 57.2	68.7 ± 66.3
*IL6*	0.07 ± 0.06	0.03 ± 0.01	0.05 ± 0.03	0.08 ± 0.05	0.14 ± 0.10	0.24 ± 0.15	0.15 ± 0.09	0.01 ± 0.00	0.05 ± 0.02	0.03 ± 0.01	0.11 ± 0.09	0.42 ± 0.38
*IL8*	0.11 ± 0.06	0.03 ± 0.01	0.03 ± 0.01	0.05 ± 0.02	0.09 ± 0.04	0.19 ± 0.10	0.14 ± 0.09	0.02 ± 0.01	0.06 ± 0.02	0.09 ± 0.06	0.07 ± 0.04	0.06 ± 0.03
*HMOX1*	2.13 ± 0.54	4.69 ± 2.01	3.86 ± 1.48	5.00 ± 1.94	4.36 ± 1.97	6.17 ± 2.65	4.07 ± 1.24	1.12 ± 0.28	7.67 ± 4.72	3.41 ± 1.15	2.95 ± 0.56	2.76 ± 0.77
*OGG1*	3.69 ± 2.37	2.47 ± 1.44	2.56 ± 1.33	3.28 ± 1.67	2.82. ± 1.54	5.17 ± 2.43	4.81 ± 1.78	1.85 ± 1.14	3.35 ± 2.08	2.65 ± 1.06	6.33 ± 3.30	6.31 ± 14.3
Adhesion molecules												
ICAM1	122 ± 3.34	123 ± 3.16	118 ± 3.40	120 ± 4.26	122 ± 15.6	119 ± 2.11	124 ± 2.56	124 ± 2.45	117 ± 2.72	122 ± 3.84	121 ± 2.82	123 ± 3.03
ITGAL	114 ± 9.13	117 ± 9.60	104 ± 16.5	130 ± 4.26	121 ± 3.49	109 ± 9.19	120 ± 9.16	111 ± 9.20	114 ± 10.8	105 ± 9.63	115 ± 10.5	123 ± 11.8
L-Selectin	227 ± 24.3	146 ± 11.4	159 ± 16.5	157 ± 11.9	234 ± 17.9	196 ± 15.1	202 ± 14.0	262 ± 31.8	223 ± 28.3	205 ± 19.4	189 ± 18.5	155 ± 13.5

*p *> 0.05 for all differences

## Discussion

This study shows that 3 h inhalation exposure to wood smoke had no apparent effect on MVF, oxidatively damaged DNA and biomarkers related to inflammation, oxidative stress and DNA repair in atopic adults.

The wood smoke in our study was generated in a stove with air supply for optimum combustion conditions of the wood. The chamber air had a bimodal particle number side distribution with mean count diameter peaks at 67 and 157 nm (Figure 1). In a study of particles emitted from a wood pellet burner, optimum combustion condition was associated with mainly inorganic particles with size corresponding to our small peak, whereas poor combustion conditions generated particles mainly composed of carbonaceous material as organic compounds and soot and with size corresponding to our large peak [25]. In the wood pellet burner study the small size particles showed considerable hygroscopic growth possibly explaining low airway deposition of wood smoke particles at both types of combustion conditions and this may apply to our study as well [25].

The MVF in this group of atopic subjects (MVF score = 1.56) was lower compared to the MVF in our previous studies with healthy non-atopic subjects (MVF score = 2.04) and elderly subjects (MVF score = 1.78) [20,22]. The assessment of MVF in healthy subjects in the present study confirmed that subjects without atopy had larger MVF (MVF score = 1.91) and the majority of the variation was attributed to inter-individual differences. A direct comparison between these healthy subjects and the atopic subjects in the wood smoke exposure study should be done with caution because the subjects were recruited in different ways. It has been shown that patients with asthma had decreased vasodilatory response to shear stress [26], although it is not known whether this had relation to atopy which is common in asthmatics. The lower level of MVF in our atopic subjects without asthma is intriguing because such low MVF score has been reported as an independent predictor of cardiovascular events among patients at risk [27]. Still, atopy appears not to have been considered as risk factor for cardiovascular health effects. More important with regard to our study is that it may have been difficult to detect a decline in the MVF after wood smoke exposure when the MVF score was low already, whereas subjects without atopy might have responded differently. Our findings concerning the MVF score are consistent with a very recent study showing no effect of exposure to wood or coal smoke (160-200 μg/m^3^) for 3 hours on MVF assessed by the EndoPAT device in 26 healthy non-smoking young adults, although elevated ambient levels of fine particles in the area in the previous 2 days was associated with low score, suggesting effects of that on the MVF [28]. Exposure to second hand smoke has also been shown to reduce MVF assessed by the EndoPAT device [29]. Moreover, it was recently shown that indoor air filtration was associated with a 60% reduction of PM_2.5 _and a 9.4% increase in MVF score assessed by the EndoPAT device in healthy subjects from a wood smoke impacted community [21]. In a similar type of intervention study, we have previously shown that filtration of indoor air in homes was associated with an 8.1% increase in MVF score assessed by the EndoPAT device in elderly individuals and this effect was associated with reduction in potassium in the particles, suggesting biomass as a relevant source [20]. These two intervention studies were of longer duration than the short term exposure applied here. On the other hand, significant effects on endothelial function assessed as forearm blood flow after intra-brachial bradykinin administration and other vascular functions have been demonstrated within 6 h after exposure to only 1 h of diesel exhaust at similar concentrations (250 μg/m^3^) but with exercise [30,31], and this is not related to nitrogen dioxide or caused by pure carbon particles [32,33]. Although assessed in different settings with different methods this apparent difference in response to similar mass concentrations might suggest that diesel exhaust particles are more potent than wood smoke particles in terms of effects on vascular function. Indeed, the airway deposition fraction of traffic-generated particles is much higher than that of wood smoke particles [34]. Moreover, mass concentration is just one simple metric, whereas wood smoke and traffic emissions are complex mixtures with many other differences in size distribution and chemical composition as well as volatile compounds and gases.

We investigated the expression of genes involved in cytokine production in PBMCs as well as surface markers of cell adhesion molecules because the activation of monocytes with inflammatory signalling and adhesion to the endothelium through integrins, L-selectin and ICAM1 or VCAM1 interactions are important steps in the atherosclerotic process [3,35]. *In vitro *particulates from poor combustion of wood increased adherence of THP-1 monocytes to human umbilical vein endothelial cells, possibly due to increased expression of *IL8 *and *TNF *in the former and increased surface expression of VCAM1 in the latter [36]. In asthmatic subjects inhalation of ultrafine carbon particles was associated with reduced expression levels of Mac-1 integrin (CD11b/CD18) on monocytes and reduced ICAM-1 on polymorphonuclear leukocytes [37]. In a cross-sectional study of Indian women, those cooking with biomass and a chronic particle exposure around 600 μg/m^3 ^showed increased Mac-1 expression on monocytes and neutrophils as compared with those cooking with liquefied petroleum gas although confounding from e.g. life-style factors cannot be excluded [38]. A 2-h lasting exposure to smoke from an electrically heated oak log with a particle concentration of 484 μg/m^3 ^and including exercise indicated an increased percentage of neutrophils in both blood and alveolar lavage fluid 20 h later [39]. Our results do not indicate expression of adhesion markers in terms of integrin αL (ITGAL), L-selectin and ICAM1 or increased expression of *CCL2, IL6, IL8, TNF *and *ITGAL *in PBMCs of atopic subjects at the time points we studied. The exposure to wood smoke particles did evoke symptoms of airway mucosal irritation [23], yet this local effect in the respiratory epithelium might not transmit to systemic inflammation or oxidative stress detectable by our biomarkers. However, in a similar study with 13 subjects exposed to wood stove smoke at 240-280 μg/m^3 ^for 4 h with light exercise for 50 min there were subtle signs of oxidative stress and inflammation in terms of increased urinary 8-isoprostaglandin-2α, serum amyloid A, alveolar nitric oxide, malondialdehyde in breath condensate and serum clara cell protein [13]. On the other hand, direct pulmonary effects seem to be minor as shown by a recent study finding increased concentration of glutathione in the respiratory tract lining fluids of the distal lung, whereas there were no other inflammatory or pulmonary effects after exposure to similar concentrations (224 μg/m^3^) of wood pellet burner smoke [40]. The total level of PAH just in PM was even higher in our study than the total level including semivolatile PAH in these two previously reported wood smoke exposure studies [40,41]. In accordance, the collected set of studies on healthy subjects exposed to wood smoke particles up to 400 μg/m^3 ^for 3-4 h showed very limited signs of systemic or local inflammation and oxidative stress with the biomarkers used so far [12,13,16]. At higher doses or concentrations such as those used in animal experiments or cell culture studies wood smoke particles can induce substantial oxidative stress and inflammatory responses [9-11,42].

We have previously observed that few hours of exposure to traffic-generated air pollution PM_2.5 _at levels of 15-25 μg/m^3 ^in Copenhagen was associated with elevated levels of oxidatively damaged DNA in PBMCs [43-45], whereas wood smoke exposure at around 260 μg/m^3 ^with light exercise generated minimal signs of possible DNA oxidation in terms of *OGG1 *upregulation in only 13 subjects [15]. In the present larger study with control and two exposure levels in random order as well as high levels of PAH we found no *OGG1 *upregulation or change in the level of oxidatively damaged DNA, which was within the reference values for human leukocytes in the present study [46]. Our quality control FPG sensitive sites in Ro19-8022 and white light treated reference samples were also within the target values in all comet assay runs. The present results support that wood smoke particles are less potent than traffic-generated air pollution particles in terms of inducing oxidatively damaged DNA. This could be partly explained by the fact that the deposited amount of traffic exhaust particles was found to be 16 times higher by number and 3 times higher by surface area compared to the deposition of particles from a wood pellet burner [25]. Furthermore, animal experimental studies showed that exposure to diesel exhaust particles by oral gavage on mass basis generated more oxidatively damaged DNA in the liver and especially lung of rats than did wood smoke particles [42,47]. Still, both type of particles generate oxidatively damaged DNA in cultured human cells with the organic fraction and low oxygen combustion conditions of wood smoke particles being most potent [9,10]. This supports that experiments in cultured cells and animal experiments can be used to identify common mechanisms of action of particle-generated oxidative damage to the DNA [7].

Our results indicate that 3-h exposure to wood smoke particles at relatively high concentrations far above ambient levels in Denmark is not associated with detectable systemic inflammation, oxidative stress, DNA damage or altered MVF in potentially susceptible subjects with atopy. For MVF the confidence interval around the effects size of the wood smoke exposure as such indicated that we should not have missed a reduction of more than 5%, which would be of limited clinical relevance. We have sufficient statistical power to detect realistic differences in the range of 15-25% differences on endpoints that previously have shown effect after exposure to traffic-generated ambient air particles. It should be emphasised that the combustion conditions are important determinants for the physiochemical properties of wood smoke particles, such as size distribution and PAH content, and this may have an impact on the toxicological properties of the exposure [1]. The subjects in our study inhaled particles of two size modes of possible inorganic ash and carbonaceous material that have shown limited deposition at similar combustions conditions, respectively [23,25]. We used optimal combustion conditions and dry beech wood as fuel, which may generate particles with low deposition fraction and limited toxicity. It cannot be ruled out that other types of wood and ineffective combustion conditions generate stronger effects. In real life, suboptimal combustion could be related to wood stoves with suboptimal design, high moisture content of the wood, or wood that is not intended for combustion in wood stoves such as pressure-treated timber and plywood. In addition, long-lasting exposure to wood smoke particles could be associated with health effects, and exposure to wood smoke may induce more effect on inflammation, oxidative stress, and MVF in high risk groups such as the elderly, whereas atopy might decrease, rather than increase susceptibility to some effects as possibly seen here for the MVF.

## Conclusions

The results of this study indicate that smoke from a well burning modern wood stove, from the presently investigated 3-h exposure scenario, has limited systemic effects in potentially susceptible atopics, as no significant oxidative stress, inflammatory signalling or MVF reduction was demonstrated.

## Materials and methods

### Subjects

Details on the design of the wood smoke exposure study and the subjective symptoms reported by the participants are reported elsewhere [23]. In brief, 20 healthy atopic non-smoking subjects were recruited, 10 men and 10 women, aged 19-55 years (median 25 years) with a mean ± SD body mass index (BMI) of 22.74 ± 2.03. Inclusions criteria for the subjects included two positive prick tests out of ten aeroallergens, and no personal history of cardiovascular disease. Prior to the study, all subjects underwent medical assessment consisting of health history, clinical examination and spirometry. Pregnancy, abnormal lung function, bronchial hyper-responsiveness, and a medical history of other diseases were exclusion criteria. Bronchial hyper-responsiveness was diagnosed if an additive provocative dose between 0.063-0.241 mg of metacholine caused a ≥ 20% decrease in FEV1. The participants were free from known infections or airway symptoms for at least 1 week before the experiments, and had taken no drugs during the 48 h prior to the exposure sessions.

In order to address possible diurnal variation we also measured MVF in the morning, before and after lunch and in the afternoon of one day in eight healthy subjects without atopy but with the same age range as the atopic subjects.

### Study design

The study was designed as a three-way cross-over study, with randomised sequence of inhalation exposure to clean air or wood smoke exposure at low or high concentrations. Each exposure lasted for 3 h and the subjects served as their own controls. For each episode four subjects were exposed simultaneously in the climate chamber in sessions separated by 14 days. The study lasted from January to June 2008. During the sessions both the subjects and investigators were blinded to the exposure. We obtained blood samples before (baseline) and at 0, 6 and 20 h after the exposure. In addition, we measured blood pressure and MVF 6 h after the exposure. Vascular dysfunction has been reported 6-8 h after exposure to diesel exhaust [30,48]. MVF was not measured at other time points because of possible effects of ischemia on biomarker measurements.

The study was approved by the local ethics committee (M-20070097) and in accordance with the declaration of Helsinki and all subjects gave written informed consent before inclusion.

### Generation of wood smoke and characterisation of exposure

Exposure sessions took place under controlled conditions in a 79 m^3 ^climate chamber [23]. We used beech wood with a relative humidity of 16-20% that was combusted in a Morsø model 7110 wood stove (Morsø Jernstøberi, Nykøbing Mors, Denmark) with a burn rate of 1.7 kg/h and air supply for maximal combustion efficiency. The target mass concentrations of wood smoke were achieved by dilution of the flue emission conditioned in an aging pre-chamber with filtered ambient air. The climate conditions in the chamber were stable, and during the exposure the temperature was 22.94 ± 0.05°C and the relative humidity stayed within the range of 22-32%. The exposure sessions were conducted between 09.00 am and 14.00 pm. The aerosol load in the chamber was built up for 30 min and then maintained at equilibrium for 180 min.

Stationary measurements of PM with diameters smaller than 2.5 μm (PM_2.5_) were carried out by gravimetric determination of mass sampled on Teflon filters during each session. A Differential Mobility Particle Sizer was used to monitor particle number size distribution during the exposure sessions in the size range from 10 to 700 nm. The total number of particles N_tot _± SD was 222 ± 358 cm^-3^, 29112 ± 11883 cm^-3 ^and 71036 ± 46471 cm^-3 ^for the clean air, low and high exposure, respectively, Table 1.

For analysis of PAH quartz microfiber filters were used to collect material as described in details elsewhere [23]. The filters were spiked with deuterium-labelled standards, extracted with dichloromethane, transferred to hexane on silica acid glass columns and analyzed for chrysene + triphenylene, benzofluoranthene, benzo[e]pyrene, benzo[a]pyrene, preylene and indeno(1,2,3-cd)pyrene by gas chromatography-mass spectrometry.

### Microvascular function

MVF was measured non-invasively using peripheral arterial tonometry during reactive hyperaemia. The properly functioning endothelium of the digital arteries should respond to the flow-induced shear stress by nitric oxide production resulting in dilation and wide amplitude in the pulse wave in the fingers. This method is increasingly used clinically for assessment of endothelial function, is reproducible, has compared favourably with most other methods and predicts coronary events beyond other risk factors [49]. Briefly, this technique uses finger-mountable pneumatic sensors (EndoPAT-2000, Itamar Medical Ltd, Cesaria, Israel) specifically designed to continuously record the digital arterial pulse wave. Baseline was measured as a steady state and during a 5 min occlusion period the signal disappears completely (supra systolic cuff inflation). This was followed by a clear increase in signal during recovery (reactive hyperaemia). The contra lateral digit recording was the control over time. All testing was performed in a quiet laboratory environment with dimmed lighting. The data were digitally stored as pulse wave tracings that were reviewed for off-line analysis using the display and measurement properties of the software. If both tracings were of good quality a MVF score was calculated as the average amplitude of the MVF signal after cuff deflation divided by the average amplitude before the inflation [20]. A MVF score ≤ 1.67 is considered as abnormal according to the manufacturer (Itamar Medical Ltd, Cesaria, Israel). MVF was computed using an automated algorithm supplied with the instrument. Blood pressure was measured directly before each measurement.

### PBMC separation

PBMCs were isolated from citrate-treated venous blood by Lymphoprep^® ^gradient and subsequently frozen at -80°C in a mixture containing 50% fetal bovine serum (FBS, GibroRBL), 40% culture medium (RPMI 1640, GibcoRBL) and 10% dimethyl sulfoxide for the comet assay and FACS analyses. PBMCs for gene expression were cryopreserved in TRIzol^® ^reagent (Invitrogen A/S, Taastrup, Denmark).

### Gene expression

The mRNA levels of *IL6*, *IL8*, *TNF*, *CCL2*, *ITGAL*, *HMOX1*, and *OGG1 *were measured in PBMCs. The PBMCs in TRIzol were rapidly thawed and the RNA was extracted and DNAse treated (Promega Biotech AB, Denmark). After reverse transcriptase-mediated cDNA synthesis quantitative real-time PCR reactions were carried out in ABI PRISM^® ^7900HT (Applied Biosystems, Naerum, Denmark) using primers and cDNA specific probes purchased from Applied Biosystems. The assay IDs for the genes were as follows: *ITGAL*, Hs01035619_m1; *CCL2*, Hs00234140_m1; *IL6*, Hs00985641_m1; *IL8*, Hs00174103_m1; *TNF*, Hs00174128_m1; *HMOX1*, Hs00157965_m1; *OGG1*, Hs01114116_gl. We used commercially available 18S rRNA from a probe and primer solution (Eukaryotic 18S rRNA Endogenous Control, 4352930E, Applied Biosystems) as a reference gene. The PCR reactions were performed as previously described [45]. The level of gene expression is reported as the ratio between the level of the target gene and the 18S rRNA reference gene using the comparative 2^- ^*^ΔCt ^*method.

### Single-Cell alkaline Gel Electrophoresis (Comet assay)

The levels of strand break (SB), endonuclease III- (EndoIII) and formamidopyrimidine DNA glycosylase (FPG) sensitive sites were detected by the comet assay as described previously [24]. The EndoIII and FPG enzymes were gifts from Professor Andrew Collins (University of Olso, Norway). Briefly, PBMCs were embedded in 0.75% low-melting point agarose (Sigma-Aldrich A/S, Brøndby, Denmark) on GelBond^®^films (Lonza Copenhagen Aps, Vallenbæk Strand, Denmark) and lysed (1% Triton X-100, 2.5 M NaCl, 100 mM Na_2_EDTA, 10 mM Tris, pH 10) for a minimum of 1 h at 4°C. The Gelbond films were then immersed in an alkaline solution (300 mM NaOH, 1 mM Na_2_EDTA, pH 13) for 40 min and the duration of the subsequent electrophoresis was 20 min at 0.83 V/cm (cathode to anode) and 300 mA. After electrophoresis the Gelbond films were washed 3 times 5 min in Tris buffer (0.4 M Tris-HCl, pH 7.5), rinsed with milliQ^® ^water and dried in 96% ethanol.

We scored nuclei with an Olympus fluorescence microscope at 40x magnification with visual inspection after staining with YOYO-1 in PBS (Molecular Probes, Eugene, OR). All samples from one subject were coded and analysed simultaneously in order to minimise inter-assay variation.

We analysed 100 comets per slide and there were four slides for each sample, corresponding to a total number of 400 nuclei. The slides had been prepared in duplicates on two different assay runs (including different electrophoresis). The nuclei were scored by visual classification based on a five-class scoring system (arbitrary score range: 0-400) as previously described [24,50]. We had reference control samples in each experiments (corresponding to one electrophoresis) that included one aliquot of undamaged PBMC and PBMC that had been exposed to the photosensitizer Ro19-8022 and white light, which generates high levels of FPG sensitive sites. The Ro19-8022 photosentizer was a kind gift from F. Hoffmann-La Roche (Basel, Switzerland). The number of EndoIII and FPG sensitive sites was obtained as the difference in scores of parallel slides incubated with and without EndoIII or FPG. These scores were transformed to lesions per 10^6 ^base pairs (bp) by means of a calibration curve based on induction of SB by ionising radiation, which has a known yield. We used a conversion factor of 0.0298 Gy equivalents per score and calculations were based on the assumption that an average molecular weight of a DNA bp is 650 Dalton. We have > 15 years experience and coordinate and participate in several comet assay validation studies, including The European Comet Assay Validation Group (ECVAG) [51,52].

### Immunofluorescence analysis of ICAM-1, ITGAL and L-selectin

The cryopreserved PBMC samples were rapidly thawed and incubated with saturating concentrations of phycoerythrin (PE)-conjugated mouse anti-human CD54 (ICAM1), fluorescence isothiocyanate (FITC)-conjugated mouse anti-human CD11a (ITGAL) and allophycocyanin (APC)-conjugated mouse anti-human CD62L (L-selectin) (Becton Dickinson (BD) Bioscience, Brøndby, Denmark) for 20 min in darkness. Cells labelled with PE, FITC, APC isotype-matched hAb served as control (BD biosciences) and to assist in appropriate gate setting. THP-1 cells treated with 10 ng/ml phorbol 12-myristate 13-acetate for 24 h served as a positive control. Fluorescence intensity of at least 10^5 ^cells per sample was analysed with a FACS Calibur (BD Biosciences). Data acquisition and processing were performed with Cell Quest software (BD Biosciences).

### Statistical analysis

Due to a few missing values we used a mixed model repeated-measure analysis, PROC MIXED procedure of SAS v8.2, (SAS Inst. Inc., Cary, NC) to analyse DNA damage (FPG, EndoIII and SB), outcome MVF, gene expression and adhesion molecules expression.

The model included sex nested in subject, subject*time and subject*exposure interactions as random effects and time according to exposure (baseline, 0, 6 or 20 h), level of exposure (clean air, low and high wood smoke concentration), previous exposure in order to assess possible carry-over effect, calendar period and time*exposure as fixed effects. All data were skewed; therefore the statistical analysis was performed on the natural logarithm of the included outcome variables. P-values < 0.05 were considered to be statistically significant. There were no significant interactions in the statistical analysis and the p-values refer to the statistical significance of the wood smoke exposure. We assessed the statistical power of the study by calculating the number of subject that would be required to detect a 10% reduction in MVF based on the residual variation in ANOVA tests in our previous studies of MVF [20,22]. We would able to detect a 10% reduction in the MVF score at 80% power with 20 subjects.

## Abbreviations

MVF: microvascular function; PBMC: peripheral blood mononuclear cells; HMOX1: heme oxygenase 1; ICAM1: inter cellular adhesion molecule 1; ITGAL: integrin αL; CCL2: chemokine (C-C-motif) ligand 2; IL: interleukin; SB: strand breaks; FPG: formamidopyrimidine DNA glycosylase; OGG1: oxoguanine DNA glycosylase-1; EndoIII: endonuclease III.

## Competing interests

The authors declare that they have no competing interests.

## Authors' contributions

IRS, JB and TS conceived and organised the human exposure. AM characterized the exposure aerosol. LF, PM and SL conceived the use of oxidative stress, inflammation and microvascular function markers and LF were responsible for all the analyses. LF and SL drafted the manuscript, which was critically revised by all authors, who have read and approved the final version.
